# Tuck-in tenon patch graft for giant full-thickness macular holes

**DOI:** 10.1186/s40942-024-00561-5

**Published:** 2024-07-01

**Authors:** Maher Saleh, Chiatse Ellalie Koman

**Affiliations:** Department of Ophthalmology, Farah Hospital Abidjan - Ivory Coast, Abidjan, Côte d’Ivoire

**Keywords:** Full, Thickness macular hole, Tenon patch graft, Conjunctiva, vitrectomy

## Abstract

**Purpose:**

To report the results of using autologous Tenon patch grafts for managing giant full-thickness macular holes (FTMHs) when other alternatives are not applicable.

**Methods:**

The same surgical technique was performed in all three cases. Briefly, a small fragment of Tenon’s tissue was collected. The graft was introduced through a 23G trocar and released over the macular hole under a bubble of PFCL. The patch is delicately pushed towards the edges of the hole to slide underneath. The PFCL bubble is then actively aspirated next to the optic disc. Tamponade with gas or silicone oil is subsequently injected, with care taken to minimize fluid turbulence during the procedure.

**Results:**

The outcomes of autologous Tenon patch grafts in three giant FTMHs are reported. In the first case, silicone oil tamponade was injected, in the second, C2F6 gas was injected. And in the third case, that of a woman with advanced glaucoma, no tamponade was left in the eye. No adverse effects were observed during or after the procedures. Closure of the macular hole and functional improvement were documented during the follow-up period in all three cases.

**Conclusion:**

With a follow-up of up to 6 months, the Tenon patch graft appeared to be a promising technique for managing complex cases of FTMH. Additional studies to investigate long-term outcomes and determine the most appropriate indications are warranted.

## Introduction

Pars plana vitrectomy with or without peeling of the internal limiting membrane (ILM), followed by intraocular air or gas tamponade, is considered the standard procedure for treating idiopathic full-thickness macular holes and has a high anatomical success rate [1, 2]. However, some cases are more challenging, particularly in the presence of a giant macular hole, underlying rhegmatogenous retinal detachment, or failure of previous surgery. Several techniques have been proposed to manage these complex situations, including neurosensory retinal grafts, the use of ILM flaps, human amniotic membrane plugs, autologous serum or platelet-rich plasma adjuvants, lens capsular flaps, or perifoveal hydrodissection [3, 4].

However, when the ILM has been previously peeled extensively, options are limited in cases of refractory macular hole, especially when an amniotic membrane is not available in the absence of eye bank support. Therefore, we devised a technique based on Tenon patch graft (TPG) to temporarily fill the macular hole. Tenon’s membrane offers several advantages: it is easily accessible and cost-effective and has minimal or no immune or infectious risk. Durable sealing is expected due to its collagen composition and thickness. In the absence of safety data on this new procedure, we relied on previous reports: Tenon’s capsule has been used in the management of scleral and corneal perforation with a good safety profile. The use of autologous tissue evokes a minimal immune response, thus eliminating any chances of tissue rejection. Furthermore, the accidental presence of epithelial cells from the conjunctiva in the vitreous cavity has been reported during vitrectomy without any consequences [5]. We present the first cases of patients who underwent this procedure.

## Methods

The same surgical technique was performed in all three cases. In practice, povidone iodine at 5% is applied twice with a 2-minute contact time on the surface of the eye. A nasal conjunctival opening is created, followed by the collection of a small fragment of Tenon’s tissue without apparent vessels. The sample is then trimmed with scissors to measure approximately 1 mm in diameter using a caliper. The graft is intentionally made slightly smaller than the MH because Tenon’s membrane tends to swell upon contact with water.

Two milliliters of PFCL are injected into the posterior cavity. The 23-gauge trocar valve in the superior position is then removed, and the Tenon’s graft is introduced using a 23-gauge ILM forceps. Visualization of the macula is achieved using a disposable vitrectomy lens (DORC, The Netherlands). The Tenon’s graft, released over the macular hole, descends to the bottom of the hole under the weight of the PFCL. With the forceps’ closed jaws, it is gently pushed towards the edges to slide underneath them when they are slightly elevated. But even if the edges are not raised, the Tenon’s capsule tends to remain in place because of its sticky consistency. The PFCL bubble is then actively aspirated next to the optic disc. Tamponade with gas or silicone oil is subsequently injected, with care taken to minimize fluid turbulence during the procedure. In the latter case, no tamponade was used.

The cases were discussed by the ethics committee of the Farah hospital’s surgical department, which approved the study contingent upon the patient’s informed and voluntary consent. Providing patients with enlightened information and appropriate medical advice was an essential step before surgery. Written consent was obtained before the procedure in all patients.

## Case series

### Patient 1: tenon patch graft under silicone oil tamponade

The first case we report concerns a 38-year-old man who underwent complex retinal detachment surgery with inferior retinal retraction (Grade C vitreoretinopathy) and a full-thickness macular hole. Following chromovitrectomy (Membrane Blue Dual (Dutch Ophthalmic Research Center International, Zuidland, The Netherlands)) with peeling of the internal limiting membrane (ILM) and silicone oil tamponade, the retina was reattached, and the hole was closed. However, after the extraction of silicone oil, the macular hole reopened and significantly enlarged to a horizontal diameter of 1211 μm and a vertical diameter of 1353 μm, triggering redetachment of the macula and extension to the inferior retina. As the amniotic membrane was unavailable due to regulatory constraints, the patient received a clear explanation of the situation. Given the risk of functional loss of the eye due to retinal detachment and the absence of alternative options, the patient consented to the procedure. In the absence of previous similar procedures or animal models, a comprehensive list of complications was provided to the patient. The risks of infection, neovascularization, and proliferative vitreoretinopathy (PVR) among others were explained.

**Fig. 1:**
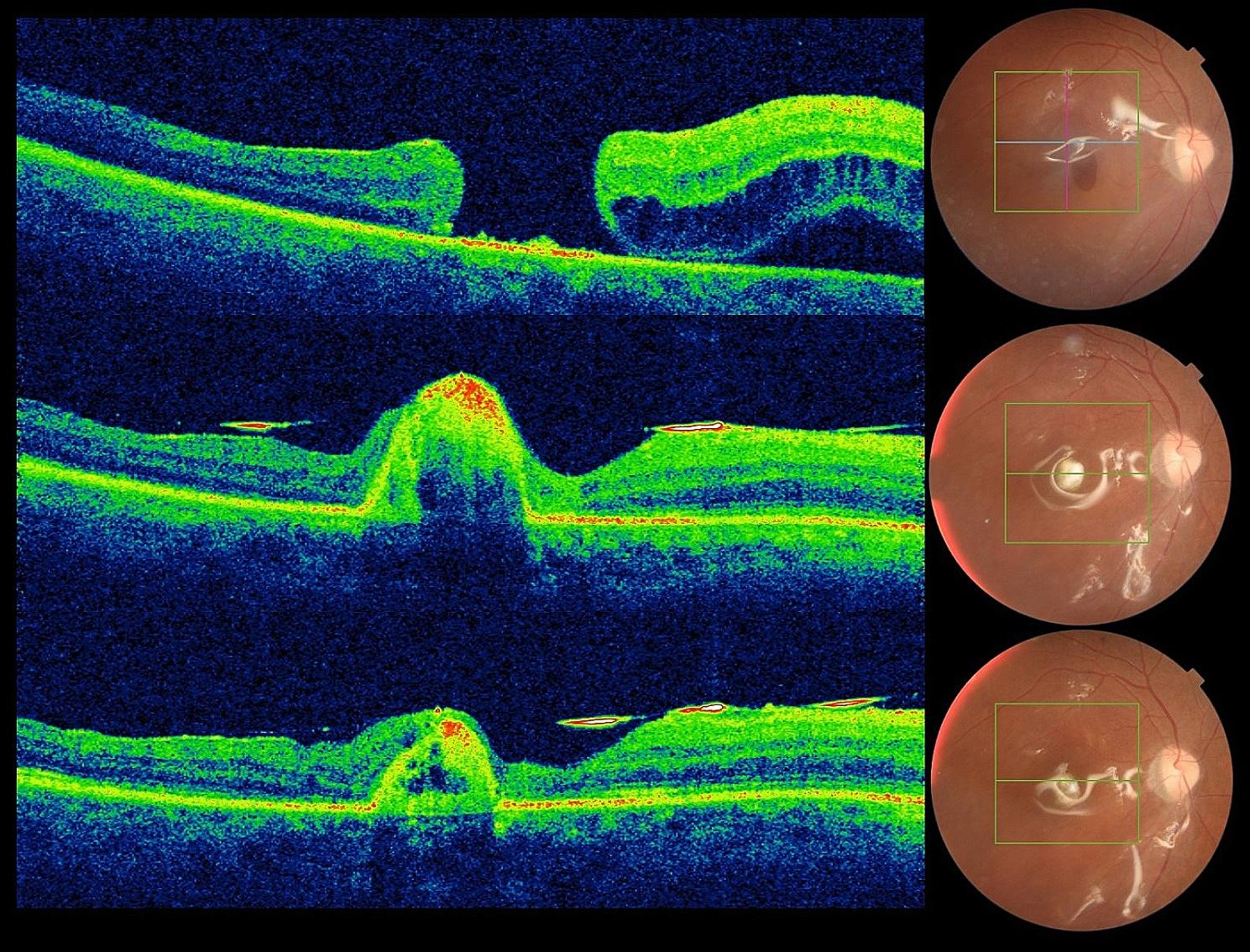
Patient 1. A horizontal B scan showing the presence of a giant full-thickness macular hole with a vertical diameter of 1353 μm associated with a detached retina (top). Postoperative B scan showing the Tuck-in Tenon Patch Graft under silicone oil. (Middle). A corresponding B scan at 5 months of follow-up showing good resorption of the patch with closure of the MH (bottom). At the last check-up, there was a noticeable decrease in the amount of Tenon’s patch, which is now located under the retina. The macular hole was closed. Inside the patch, small cavitations are observed, the histopathological significance of which is not known. The associated OCTA (not shown) did not reveal any associated neovascularization

To ensure infection control, 5% povidone iodine was instilled twice at the collection site. The graft was harvested via the same procedure. The PFCL was extracted, and the posterior segment was filled with 1000 cSt silicone oil. The Tenon patch graft was monitored during postoperative visits using SD-OCT, which revealed progressive shrinkage of the graft. The retina remained attached, and the sealing effect of the graft persisted after 6 months. The best-corrected visual acuity improved from hand motion to 20/400 post operatively (Fig. 1).

### Case 2: tenon patch graft under gas

**Fig. 2:**
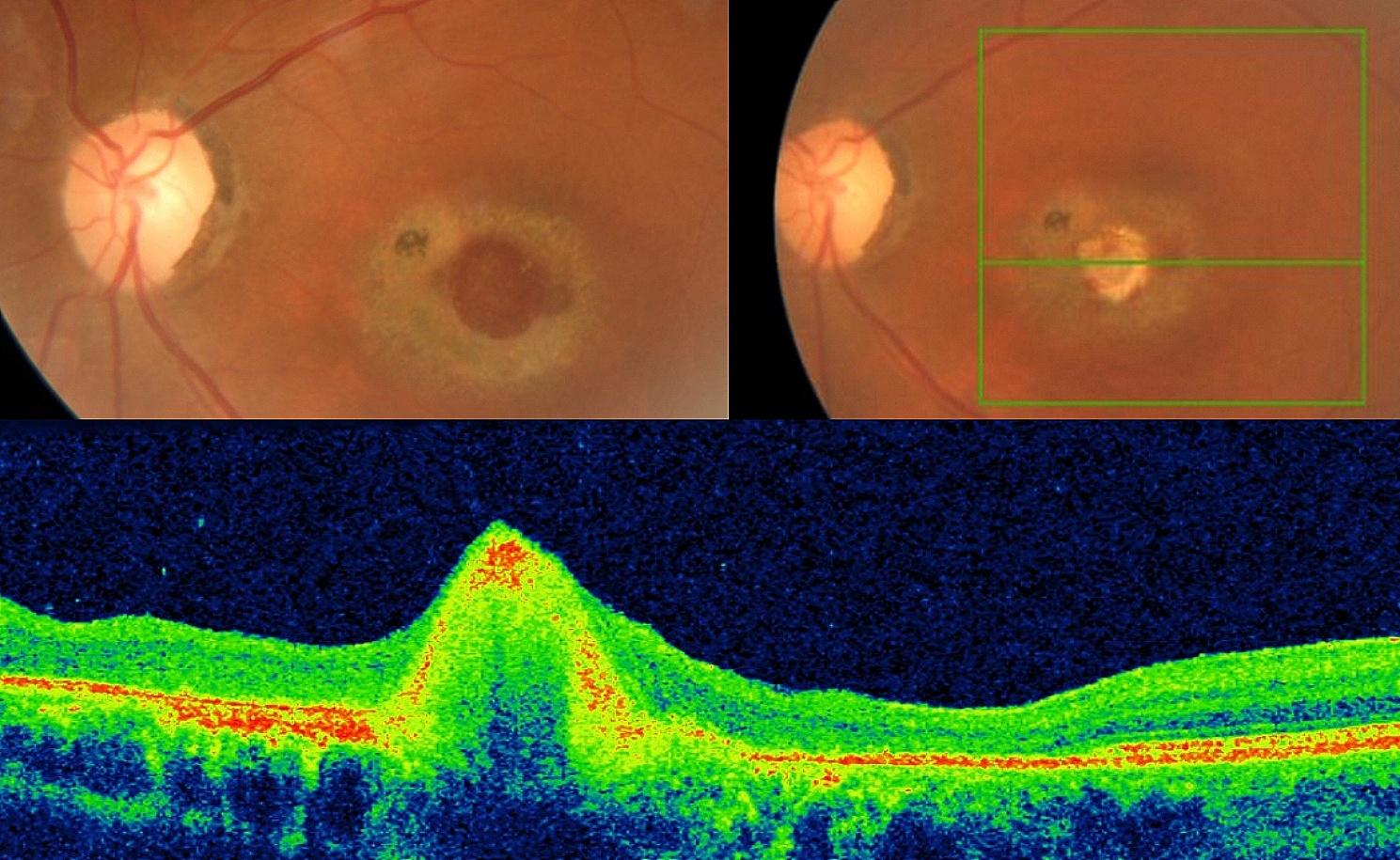
Patient 2. Upper left: Preoperative full-thickness macular hole with perifoveal atrophy related to the duration of the hole (8 years). Upper right: Tenon patch graft filling the hole. Bottom: Postoperative B scan after complete gas resorption showing the good sealing effect of the Tenon patch graft

The second patient involved a 65-year-old woman with a giant macular hole measuring 1100 microns and lasting for 8 years with marked perifoveal atrophy. The Tenon graft was harvested during the same procedure following the central vitrectomy phase, which was completed by peripheral vitrectomy under indentation. After the graft was positioned, fluid-air exchange was performed before the injection of gas (20% C2F6). After the resorption of the gas bubble, the best-corrected visual acuity improved from hand motion to 20/200 with eccentric viewing at 6 weeks post operation. The Tenon Patch Graft remained in place. Follow-up visits revealed a progressive decrease in size of the graft, and the macular hole remained closed. No adverse effects, such as inflammation, neovascularization, increased intraocular pressure, or vitreoretinal proliferation, were observed during the follow-up (Fig. 2).

### Patient 3: tenon patch graft without any tamponade

**Fig. 3:**
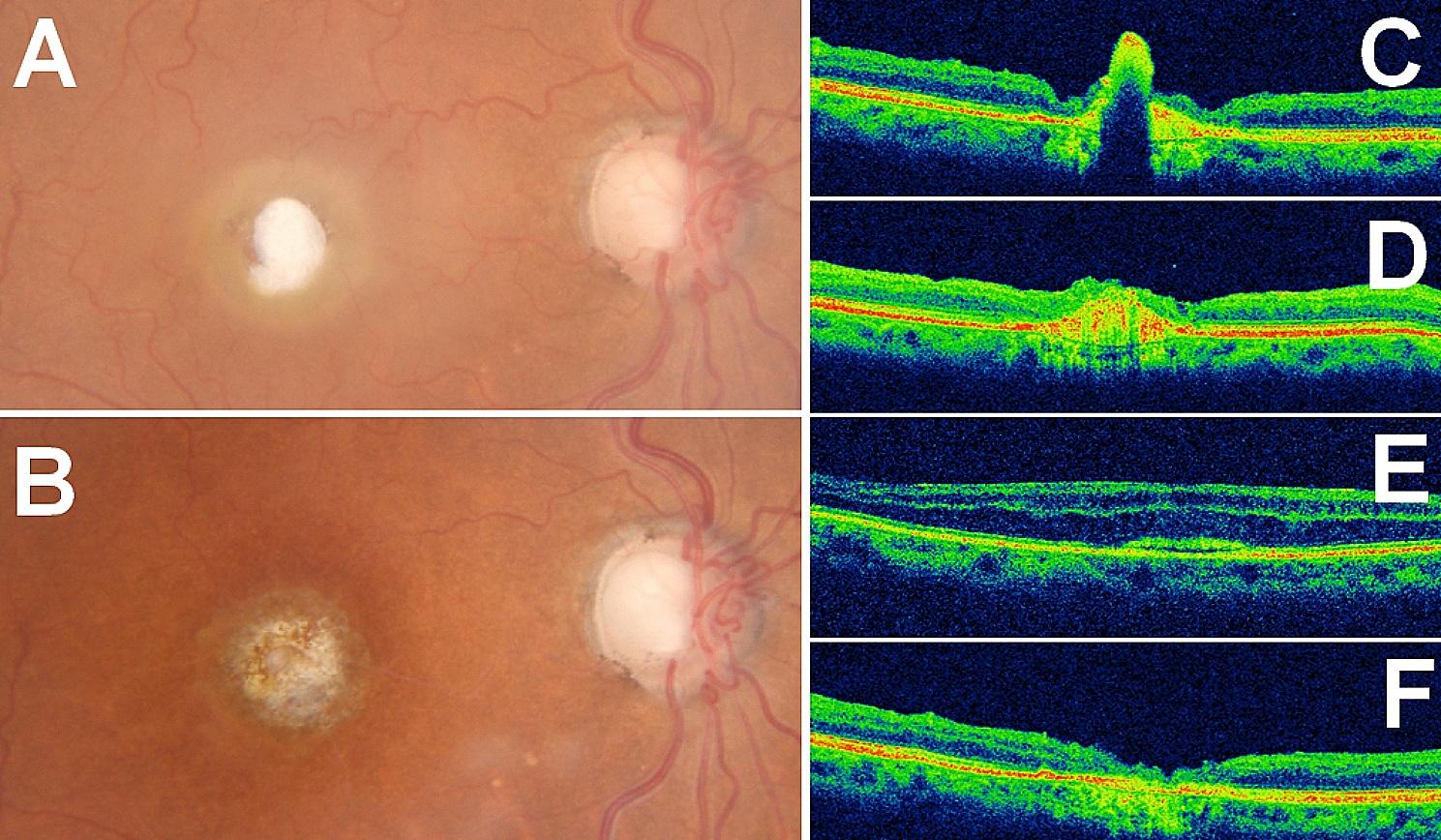
Patient 3. Full-thickness macular hole with a horizontal diameter of 1107 μm. (**A**) Tenon patch graft appearance at 1 week without resorting to any tamponade; (**B**) appearance at 3 months. (**C**) OCT image at 1 week, and at 3 months (**D**) Graft’s remnant appears as an avascular fusiform hyperreflective band. (**E**) Upper retina at the edge of the MH at 1 week and at 3 months (**F**) A decrease in retinal thickness (from 243 μm to 145 μm) was observed together with the disappearance of intraretinal cysts and a flattening of the edges of the macular hole

The third patient concerns a 73-year-old female patient who presented with long-standing visual impairment in the only functional eye. She had dense cataract as well as a giant macular hole measuring 1107 μm horizontally. Due to advanced glaucoma, she opted against internal tamponade to avoid a postoperative increase in intraocular pressure. Consequently, she underwent cataract surgery under peribulbar anesthesia followed by the harvesting of a small Tenon’s patch from the nasal area, as previously described. After the aspiration of the PFCL bubble, the Tenon’s graft was left tucked-in the macular hole and no tamponade was left in the eye. At one week, the patch was found lining the macular hole. By one month, the best-corrected visual acuity improved from hand motion to 20/100 with eccentric fixation. OCT revealed a similar fusiform appearance of the graft’s remnant in the center of the MH, with a notable decrease in perifoveal retinal thickness and disappearance of all surrounding intraretinal cysts visible on preoperative OCT. Figure 3 No complications were observed during the 6-month follow-up period, with the intraocular pressure remaining stable under triple topical therapy.

## Discussion

Tenon’s capsule is a dense, elastic, and fibrous connective tissue composed of two independent components: a thick anterior fibrous tissue with smooth muscle fibers and a fine fibrous posterior capsule in contact with orbital fat [6]. The anterior Tenon’s capsule is easily accessible, and its average thickness is 234µ [7] which means that it is thicker than an internal limiting membrane or an amniotic membrane, facilitating its manipulation. The aim of the plug was not to just fill the gap but rather to help closure, or at least reapplication, of the macular hole edges. The Tenon’s graft technique for refractory MHs is to be compared to the autologous retinal transplant, each having its own advantages and disadvantages. The autologous retinal transplant technique has demonstrated a high success rate in cases of refractory MH. Grewal and Mahmoud [8] reported an MH closure rate of 87.8% (36 out of 41 eyes). The safety profile was favorable, with major postoperative complications being retinal detachment and vitreous hemorrhage. Some authors suggest that a full-thickness retinal graft may offer improved tissue integration and potential ectopic synaptogenesis [9], potentially leading to better anatomical and functional outcomes. However, the technique involves retinal dissection with vertical scissors, endodiathermy of the borders, and the use of silicone oil as a tamponade. Sergio Rojas-Juárez et al. [9] reported a lower success rate, absence of visual improvement, with some retinal grafts not showing signs of tissue integration at any point and eventually becoming dislodged. The risk of graft loss is less problematic with Tenon’s graft given that the technique is less invasive and can be easily repeated.

In the first patient, an estimated 25% decrease in Tenon graft volume per month was observed over a 6-month period (Fig. 1). During the resorption phase, some cystic spaces were observed in the inner part of the patch with no signs of neovascularization or exudation on OCT. The structural OCT aspect of the graft’s remnant was an avascular fusiform hyperreflective band that was also avascular (OCTA not shown).

One of our concerns was that the plug would fill the macular hole without providing any anatomical or functional benefit other than preventing the occurrence of retinal detachment. Nevertheless, all patients reported noticeable and quantifiable functional improvement. In the first case, the graft melted, and the remaining part was covered by the retina, allowing the edges to join. In the last case, the edges were too far apart to fuse; however, the beneficial effect of the procedure on the surrounding functional retina was evident, with a significant decrease in retinal thickness of the surrounding retina and disappearance of the intraretinal cysts present preoperatively. Further analysis using microperimetry would likely be interesting to quantify this improvement. No safety issues were reported. During the control visit, a comprehensive eye examination was performed to detect any inflammation, increase in intraocular pressure, or signs of proliferative vitreoretinopathy. Additionally, OCT-Angiography and structural SD-OCT were conducted to detect any neovascularization. None of the eyes showed choroidal neovessels. Regarding compatibility, it involves an autologous graft, which means taking tissue from one site of a patient and transplanting it to another site within the same patient. This approach avoids issues between donor and recipient. The last case is also interesting because it represents the first successful attempt at FTMH closure without resorting to any tamponade, which was greatly appreciated by this patient who had a functional single eye. The place of this technique in our surgical arsenal remains to be determined. Its use in macular holes of common dimensions (< 800 μm) does not seem to be of interest given the high success rate of existing techniques. It appears that this technique should be reserved for very large and/or refractory MHs that are not closed by other techniques.

## Conclusion

With a follow-up of more than 6 months, the Tenon patch graft appears to be a promising technique for treating complex cases of full-thickness macular holes when other alternatives are not applicable. Additional studies will be needed to evaluate long-term outcomes and more precisely define the appropriate indications.

## Data Availability

No datasets were generated or analysed during the current study.
